# Early Treatment Consideration in Patients with Hepatitis B ‘e’ Antigen-Positive Chronic Infection: Is It Time for a Paradigm Shift?

**DOI:** 10.3390/v14050900

**Published:** 2022-04-26

**Authors:** Apostolos Koffas, Lung-Yi Mak, Upkar S. Gill, Patrick T. F. Kennedy

**Affiliations:** 1 Barts Liver Centre, Centre for Immunobiology, Blizard Institute, Barts and The London School of Medicine and Dentistry, Queen Mary University of London, London E1 2AT, UK; a.koffas@qmul.ac.uk (A.K.); l.y.mak@qmul.ac.uk (L.-Y.M.); u.gill@qmul.ac.uk (U.S.G.); 2 Department of Medicine, School of Clinical Medicine, The University of Hong Kong, Hong Kong

**Keywords:** HBeAg-positive chronic hepatitis B, HBeAg-positive chronic infection, immune tolerant disease phase

## Abstract

Chronic hepatitis B (CHB) is associated with significant morbidity and mortality, due to the adverse sequelae of cirrhosis and hepatocellular carcinoma (HCC). To date, antiviral therapy has been reserved for patients with ostensibly active liver disease, fibrosis or cirrhosis, and/or increased risk of HCC. Historically, patients with hepatitis B ‘e’ antigen (HBeAg)-positive chronic infection, were not offered antiviral therapy. Nevertheless, there has been compelling evidence emerging in recent years, demonstrating that this disease phase is in fact not characterized by immunological tolerance. HBV integration into the human genome is a frequent event found in these patients. Additionally, it may well be associated with active inflammation and fibrosis, even in the presence of persistently normal liver enzymes. Likewise, it appears that the mechanisms of hepatocarcinogenesis are already present during this early stage of the disease. This was reflected in the European Association for the Study of the Liver (EASL) guidelines, where treating patients above the age of 30 years with HBeAg-positive chronic infection was proposed. Lowering the treatment threshold to broaden treatment eligibility is likely to slow disease progression and reduce the risk of developing HCC. The current review discusses the reasons to consider early antiviral therapy in HBeAg-positive chronic infection.

## 1. Introduction

An estimated 296 million individuals are living with hepatitis B virus (HBV) globally, across developing and developed regions, the former being more profoundly affected. Despite considerable advances made in recent years in decoding and managing infection, chronic hepatitis B (CHB) remains a leading cause of morbidity and mortality, primarily due to the adverse sequelae of cirrhosis and/or the development of hepatocellular carcinoma (HCC). In particular, CHB represents the main cause of primary liver cancer worldwide; and deaths associated with CHB rose to >880,000 in 2015 [1,2].

In 2016, the World Health Organization’s (WHO) World Health Assembly (WHA) called for global elimination of viral hepatitis by 2030 and set global targets of achieving 90% reduction in new cases of hepatitis type B and C, a 65% reduction in deaths from hepatitis B and C, and treatment of 80% of people living with these infections [3]. This strategy consists of preventive measures with global vaccination programs, diagnostic interventions, and treatment goals (antiviral treatment of 80% of those eligible for treatment) [3,4]. Historically, treatment eligibility was determined by disease activity, largely based on HBV DNA level and elevated liver enzymes, in addition to the presence or absence of liver complications. However, emerging evidence suggests that treatment candidacy can be broadened, and antiviral therapy may be considered earlier during the course of infection [5,6,7]. 

The present review attempts to provide the evidence why early treatment should prevent disease progression, decrease morbidity, and mortality associated with CHB; thus, early therapeutic intervention could contribute to a decrease in the incidence and prevalence of CHB, and ultimately provide the impetus to achieve the WHO global goals.

## 2. Methods and Materials

An extensive bibliographical search was performed via the online databases PubMed, MEDLINE, Embase, and Cochrane. More recent publications from the last 5 years and highly referenced older references were prioritized. The keywords used were the following: ‘hepatitis B ‘e’ antigen (HBeAg)-positive chronic hepatitis B’, HBeAg-positive chronic infection’ and ‘immune tolerant phase’. All selected studies were manually examined to identify further relevant reports. This review included original research papers published in full, and only those written or translated into English were included. 

## 3. Immunopathogenesis and Clinical Categorization of Chronic Hepatitis B

As HBV is a non-cytopathic virus, liver inflammation that ensues is mainly contributed by the host immune response. It is accepted that the innate immune response is involved in the control and containment of acute infection, but more recently it has been recognized that innate immunity plays an important role throughout the course of CHB. Innate immune cells, through release of cytokines, chemokines, and interactions with other cells may shape the adaptive immune response [8]. It is understood that a robust virus-specific CD8^+^ T cells response is required to mediate resolution of the infection through killing infected hepatocytes and cytokine production, leading to viral clearance. In patients with CHB, on-going activation of CD8^+^ T cells and the impact of a high antigenic burden lead to their eventual exhaustion. This exhausted T cell response is the hallmark of chronic infection, resulting in a tolerogenic liver environment and consequently the inability of the host immune response to clear the virus. More recently, the importance of the role of B cells in CHB has emerged. Dysfunctional atypical memory B cells are expanded in patients with CHB, which may also limit host immune control [9,10]. Virus specific and bystander cells become activated in an attempt to control the infection, but the signals for this are poorly regulated and, thus, the homeostatic balance becomes dysregulated, leading to a prolonged inflammatory response [11]. This ultimately contributes to disease progression, the development of liver fibrosis, cirrhosis, and in some individuals, HCC [8,12,13,14,15].

Historically, the CHB disease phases were labelled as follows: ‘immune tolerant’ (IT), ‘immune active (IA)’ or ‘immune clearance’, ‘inactive carrier’ and ‘immune escape’ according to alanine aminotransferase (ALT) level, viral load, and degree of liver damage [5,6,7]. However, in 2017, the European Association for the Study of the Liver (EASL) presented a new nomenclature of the disease phases, based upon the two main aspects of chronicity, i.e., infection versus hepatitis: (i) HBeAg-positive chronic infection, (ii) HBeAg-positive chronic hepatitis, (iii) HBeAg-negative chronic infection, and (iv) HBeAg-negative chronic hepatitis [5,6,7]. CHB is a dynamic disease, which progresses through these four distinct phases in a non-linear fashion, as a result of the continuous interaction between the host immune response and the virus.

## 4. Management of Chronic Hepatitis B

Treatment of CHB primarily aims to prevent the morbidity and mortality associated with the infection [5,6,7]. Since HBsAg is believed to impair both innate and adaptive immunity, a sustained reduction in the serum hepatitis B surface antigen (HBsAg) may facilitate the recovery of the host immune response, and consequently decrease the associated morbidity and mortality [16,17,18,19]. Therefore, a functional cure defined as sustained HBsAg loss and undetectable HBV DNA, represents a realistic and achievable treatment endpoint, which is endorsed by all the major scientific societies and governing bodies [5,6,7].

Currently, treatment is indicated primarily for patients at risk of unfavorable disease outcomes, and specifically, eligibility for antiviral therapy is determined on the basis of active inflammation, established fibrosis or cirrhosis, and the risk for the development of HCC. Consequently, most guidelines recommend antivirals for patients that exhibit signs of significant or progressive liver disease. Historically, it was assumed that HBV infection remains largely harmless for long periods, until the emergence of active disease [5,6,7]. In analogy to this, HBeAg-positive chronic infection was also considered a ‘benign’ disease phase, hence, lacking criteria for consideration of antiviral treatment. International guidance on treatment of patients with HBeAg-positive CHB is summarized in Table 1.

## 5. Is HBeAg-positive Chronic Infection a Truly Benign Disease Phase?

HBeAg-positive chronic infection is characterized by high viral load, normal liver biochemistry, and the absence of or minimal inflammation/fibrosis. Historically, patients with HBeAg-positive chronic infection were excluded from treatment. This view, however, has been challenged by the recent emergence of new data, primarily on the immunopathogenesis of CHB.

### 5.1. Evidence of Immune Activity

We have demonstrated that young patients with HBeAg-positive chronic infection exhibit non-inferior HBV-specific T cell responses when compared to their peers with chronic active hepatitis. In particular, we demonstrated that young subjects with CHB have a higher frequency of HBV-specific T cells with the ability to proliferate and produce cytokines than adult patients with CHB. We also found that PD-1+ CD8 T cells, a surrogate of immune activity, were similar between patients with HBeAg–positive chronic infection and HBeAg-positive chronic hepatitis in young aged-matched subjects [20]. More recently, Traum et al. attempted to gain a deeper insight into the immune microenvironment in the HBV-infected liver and age-dependent differences in HBV immune pathogenesis. Taking advantage of the advances in the field of high-dimensional tissue imaging, which enables detailed visualization of a complex tissue microenvironment; and, in particular, imaging mass cytometry (IMC), they utilized IMC to simultaneously detect 30 immune, viral, and structural markers in liver biopsies from patients with HBeAg-positive CHB (both chronic hepatitis and chronic infection). A key finding in their study was that hepatic densities of most adaptive and innate immune subsets showed remarkably close correlations with each other (e.g., between CD4^+^ and CD8^+^ T cells, T cells and B cells, T cells and Kupffer cells, B cells, and Kupffer cells), with further correlations in their activation and/or effector phenotypes. Furthermore, hepatic densities of most adaptive and innate immune subsets correlated significantly with serum ALT. Interestingly, although patients in the ‘IA’ phase demonstrated higher proportions of HLA-DR expression (signifying an activated phenotype) among intrahepatic T cells, this marker was observed in over 50% of intrahepatic CD4^+^ cells, B cells, and Kupffer cells in ‘IT’ patients [21]. These findings provided evidence that HBeAg-positive chronic infection was not in fact associated with immunological tolerance, as previously believed. 

### 5.2. Early Integration Events

HBV DNA integration into the human genome is a frequent event found in all disease phases, including those with HBeAg-positive chronic infection [22,23]. In the historical study by Kam et al, and considering the relative paucity of data at that point in time, the researchers used cloned HBV DNA to probe for viral DNA in the liver and serum of asymptomatic CHB patients. Among four patients with HBeAg-positive disease and high viral load, they were able to detect non-integrated DNA in the liver samples, and integrated HBV DNA in one instance [22]. More recently, Rydell et al, attempted to develop a digital PCR assay discriminating between circular and integrated HBV DNA, and to relate the distribution between the two forms to other HBV markers. HBeAg-positive and -negative patients were included. They calculated that the fraction of integrated HBV DNA out of the total intra-hepatic HBV DNA was 46% in HBeAg-positive patients and that the number of integrations per hepatocyte was 1.7 in HBeAg-positive individuals [23].

More recently, Larsson et al. investigated integrations in HBV patients without HCC, representing different stages of CHB. They extracted DNA from liver biopsies of 74 patients with CHB infection and conducted analysis by Alu-PCR. Integration was detected in 39 biopsies (52%). It is noteworthy that although only three patients in this study were defined as HBeAg-positive chronic infection, all had detectable integrations. Interestingly, approximately 60% of the HBV integrations were found in noncoding regions of the human genome [24].

### 5.3. Age-Related Decline of Virus-Specific T Cell Populations

We also assessed the effect of HBsAg and the duration of infection on the host immune response, more specifically the HBV-specific T cell population. The multivariate linear regression model identified patient age as the only factor significantly associated with numbers of HBs-specific T cells (*p* = 0.000115). In patients younger than 30 years, HBs-specific T cells constituted 28.26% of the total HBV-specific T cells; and this value decreased to 7.14% in patients older than 30 years. Although the presence of HBs-specific T cells might not be required for the clearance of HBV infection in all patients, we believe that strategies to capitalize on a more functional immune response and to restore anti-HBV immune responses should be considered earlier in the course of disease and ideally, in patients under the age of 30 years [25].

### 5.4. Discordance between Serum Markers and Liver Histology

The other question that emerges with such patients, relevant to whether HBeAg-positive chronic infection is a truly benign disease phase, is whether liver enzymes and HBV DNA level can accurately predict histology. Studies have reported conflicting histological findings in HBeAg-positive patients with normal ALT and HBV DNA > 2 × 10^6^ IU/mL; some have reported mild disease on liver biopsy [26,27], while others have reported more significant pathology [28]. 

In a study of 1487 incidentally diagnosed asymptomatic HBsAg positive subjects from India, 603 (43.5%) were HBeAg-positive, and of those, 73 (5.3%) patients had persistently normal ALT (i.e., at least 3 ALT values recorded in the previous year prior to baseline liver biopsy). Of those patients with HBeAg-positivity and persistently normal ALT, interestingly, 39.7% had significant fibrosis and 63% had significant necro-inflammatory activity. The fibrosis scores among HBeAg-positive patients with persistently normal ALT were comparable between patients with HBV DNA levels ≥2 × 10^6^ IU/mL and levels of <2 × 10^6^ IU/mL (*p* = 0.649) [29,30].

In a recent prospective multi-center study, Chang et al. assessed whether patients with HBeAg-positive chronic infection may have evidence of histological liver injury, defined by Ishak fibrosis stage ≥ 3 and/or histologic activity index ≥ 9. Interestingly, it was shown that almost one in three of such patients had evident histological liver injury (EHLI). In particular, among the 181 patients with HBeAg-positive chronic infection, 60 patients had EHLI; and the proportion of significant fibrosis was higher than that of significant inflammation (33% vs. 8%, *p* < 0.001). In fact, the authors developed a nomogram to attempt to accurately identify HBeAg-positive chronic HBV infection patients with EHLI, which may respond well to antiviral therapy [31].

A recent metanalysis estimated the prevalence of fibrosis status including non-fibrosis, significant fibrosis, advanced fibrosis, and cirrhosis throughout the natural course of CHB. In total, 33 studies were included, comprising 9377 adult participants. Notably, the estimated prevalence of significant fibrosis, advanced fibrosis, and cirrhosis was for HBeAg-positive chronic infection: 16.9% (95% CI 7.8–26.1), 5.4% (95% CI 0.0–11.2), and 0.0% (95%CI 0.0–1.5), respectively. The authors concluded that fibrosis risk persists throughout the natural course of CHB [32]. These studies provide a growing body of evidence that HBeAg-positive disease may well be associated with inflammation and fibrosis, even in the presence of persistently normal liver enzymes.

## 6. The Risk of Hepatocellular Carcinoma in Chronic Hepatitis B

### 6.1. Integration as a Pilot Event in Hepatocarcinogenesis

HBV DNA integration and clonal hepatocyte expansion are considered initiating events for hepatocarcinogenesis. HBV integrants frequently involve sequences corresponding to the X gene, viral regulatory elements, and target host genes that are involved in cell cycle e.g., CTNN1, as well as tumor suppressor genes, e.g., TP53. HBV integrants often also contain sequences corresponding to the S gene [33]. Recently, Péneau et al. aimed to precisely characterize HBV integrations, in association with viral and host genomics in addition to clinical features. They showed that integration of a viral enhancer nearby a cancer-driver gene may lead to a strong overexpression of oncogenes. Secondly, their work also identified frequent chromosome rearrangements at HBV integration sites leading to downstream cancer-driver genes alterations. Notably, HBV integrations had a direct clinical impact as HCC with a high number of insertions developed in young patients and was also associated with a poor prognosis [34].

In 2016, we demonstrated that these events (HBV DNA integration and clonal hepatocyte expansion) were similar between patients with HBeAg–positive chronic infection and HBeAg–positive chronic hepatitis. HBV DNA integration and clonal hepatocyte expansion were present across all stages of CHB; and the average hepatocyte clone sizes in both early and late phases were beyond those predicted for normal liver turnover, a finding that we concluded was indicative of ongoing immune-mediated liver injury [35]. Similarly, Tu et al. showed that clonal expansion of hepatocytes with a selective advantage occurs during all stages of CHB [36]. These findings underline that events associated with tumorigenesis are already present during the early phases of chronic infection, suggesting a potential benefit with early treatment, to avert disease progression and the complications of CHB over time.

### 6.2. Substantial Risk of HCC in Patients with Untreated HBeAg-Positive Chronic Infection

The biological gradient between HBV DNA and risk of HCC is a well-known concept as demonstrated by the REVEAL-HBV study [37]. Kim et al. examined the long-term risk of HCC, mortality, and liver transplantation in subjects with no evidence of cirrhosis and HBeAg-positive chronic infection compared with patients with HBeAg-positive chronic hepatitis treated with nucleot(s)ide analogues (NUCs). The 10-year estimated cumulative incidence of HCC (12.7% vs 6.1%; *p* = 0.001) and death/transplantation (9.7% vs 3.4%; *p* < 0.001) were significantly higher in HBeAg-positive chronic infection than in the treated chronic hepatitis group. The authors argue that patients in the HBeAg-positive chronic infection phase may ultimately benefit from early antiviral therapy, especially in the context of the current generation of antivirals, such as entecavir (ETV) and tenofovir disoproxil fumarate (TDF), and more recently, tenofovir alafenamide (TAF) [38]. 

In 2019, Sinn et al. studied the incidence of HCC in those outside of current treatment recommendations and risk factors associated with HCC development; a multi-center, retrospective cohort of 3624 patients who were monitored without antiviral treatment was analyzed. The 5-year cumulative HCC incidence rate was 6.1–7.3% for non-cirrhotic CHB patients, with elevated HBV DNA levels plus mildly elevated alanine aminotransferase levels. Among patients who were outside current treatment recommendations, HBeAg-positivity, among other factors such as age, sex, cirrhosis, ALT and platelet levels, was an independent factor associated with HCC development [39].

In a large multi-cohort study that included 3625 patients, Wandeler et al. examined the incidence and risk factors of HCC among a unique subpopulation of CHB patients, co-infected with HIV, and treated with TDF-containing anti-retroviral therapy. The incidence rate of HCC remained stable over time when patients used TDF (adjusted incidence rate ratio (aIRR) per additional year: 0.95, 95% CI 0.85–1.06), whereas it increased steadily during the time patients remained treatment naive (aIRR: 1.14, 95% CI 1.07–1.21) [40]. Similarly, another study recently assessed the predictors of HCC in a multicohort study of individuals co-infected with HIV/HBV. Among 8354 individuals co-infected with HIV/HBV, 115 HCC cases were diagnosed over 65,392 person-years (incidence rate, 1.8 (95% CI, 1.5–2.1) events/1000 person-years). HBV suppression with HBV-active anti-retroviral therapy (ART) for ≥1 year significantly reduced HCC risk (aHR, 0.42 (0.24–0.73)) [41]. Although the studies involved patients with HIV/HBV co-infection; the evidence would suggest that early introduction of TDF-containing ART appeared to reduce HCC risk.

Shim et al. used a novel prediction method to estimate the risk of HCC in the Korean population based on various treatment guidelines. The 5-year risk of HCC following antiviral therapy was calculated using an HCC risk prediction model. The antiviral indications tested were the Korean National Health Insurance (NHI), the 2012 EASL guidelines [42], in addition to a “broadened” treatment indication (serum HBV DNA > 2000 IU/mL regardless of serum ALT level). Over a 5-year period, 2725 HCC cases (out of 993,872 subjects infected with HBV) were predicted per 100,000 persons (0.55%/year). When the cohort was treated based on the Korean NHI, EASL recommendations, and the “broadened” treatment indications, HCC risk decreased to 2531 (−7.1%), 2089 (−23.3%), and 1122 (−58.8%) cases per 100,000 persons, respectively (*p* < 0.0001). Based on these findings, it was suggested by the researchers that the simulated risk prediction would imply that broadening antiviral treatment candidacy may reduce the HCC risk in the general population [43].

More recently, Choi et al. conducted a multi-center cohort study including 2073 TDF- or ETV-treated, HBeAg-positive, non-cirrhotic, adult CHB patients with baseline HBV DNA levels ≥ 5.00 log10 IU/mL at three Korean centers. The researchers evaluated the on-treatment incidence rate of HCC by baseline HBV DNA levels. During the follow-up period when the patients have been on continuous antiviral treatment, 47 patients developed HCC (0.39 per 100 person-years). By Kaplan–Meier analysis, HCC risk was the lowest in those with baseline HBV DNA levels ≥ 8.00 log10 IU/mL, increased incrementally with decreasing viral load, and was the highest with HBV DNA levels 5.00–5.99 log10 IU/mL (*p* < 0.001). By multivariable analysis, baseline HBV DNA level was an independent factor that was inversely associated with HCC risk. The authors hypothesize that a decreasing but considerable viral load (e.g., 5–8 log10 IU/mL) may indicate the progressive damage of hepatocytes, clonal hepatocyte repopulation, and subsequent increased risk of HCC, compared to their peers that received early treatment with the same range of HBV DNA levels. They concluded that early initiation of antiviral treatment with a high viral load (≥8.00 log10 IU/mL) may maintain the lowest risk of HCC in those patients [44].

In contrast to these findings, Jeon et al. assessed and compared the risk of HCC in HBeAg-positive chronic infection, stringently defined by a low fibrosis-4 (FIB-4) index, in addition to high viral load and normal liver biochemistry. The authors concluded that the risk of HCC was negligible during this disease phase, in comparison to their peers with similar disease characteristics, already established on antiviral treatment [45]. Similarly, Lee et al. assessed a cohort of nearly 1000 patients in ‘immune tolerant’ phase and found that the 10-year cumulative HCC incidence was 1.7% and the 10-year cumulative incidence of phase change was 70.7%; they also reported that multivariate analysis showed that HBV DNA > 10^7^ IU/mL was associated with a reduced risk of phase change. The authors concluded that there is an extremely low risk of HCC development in patients with HBeAg-positive chronic infection [46]. Despite these findings, one can argue that the 1.7% 10-year cumulative incidence is not negligible, considering that many of these patients are young at the time of diagnosis with disease that may last for several decades if not a lifetime. This risk is expected to increase as CHB does not resolve after 10 years; and in fact, the cumulative risk should be expected to grow with advanced age and the associated emergence of co-morbidities with age, including but not limited to insulin resistance and fatty liver disease. Since it is accepted that antiviral therapy can reduce this risk further below the estimated 1.7% cumulative incidence of HCC, this begs the question—why would we exclude these patients from treatment consideration if we are trying to reduce the complications of CHB?

### 6.3. Effect of Antiviral Therapy on Viral Integration

Interestingly, Chow et al. studied 28 people who were treated with NUCs for chronic HBV infection, 14 of whom had HBeAg-positive CHB. All patients had a baseline liver biopsy and a repeat biopsy after one year of treatment; and five patients also had a third biopsy 10 years after the initiation of antiviral treatment. The investigators used inverse PCR to detect HBV DNA integration in chromosomes and expressed extent of integration as hepatocyte clone size. Prior to the initiation of treatment, all participants had detectable HBV DNA integration and a median hepatocyte clone size of 1.40 × 10^5^. After one year of NUC therapy, median hepatocyte clone size dropped by 42.5% to 6.72 × 10^4^ (*p* = 0.003). Notably, 3/5 patients who underwent a further biopsy 10 years after the initiation of therapy had no detectable HBV DNA integration in any chromosome. In these three subjects, median hepatocyte clone size fell significantly from pre-NUC therapy to year 1 and to year 10 (4.54 × 10^4^ to 2.62 × 10^4^ to < 1.00 × 10^2^, *p* = 0.015). In the five subjects who underwent three biopsies over 10 years, HBV DNA in the liver fell by 1.5 log (about 32-fold) after one year of NUC therapy and by 3.0 log (1000-fold) after 10 years. The researchers concluded that NUC therapy significantly lowered clone size of infected liver cells, and that long-term therapy significantly reduced HBV DNA integration [47]. 

More recently Hsu et al. assessed the effect of viral inhibition on transcriptionally active HBV-host integration events and explored the correlation of viral integrations with host gene dysregulation. Patients with HBV DNA > 2000 IU/mL and minimally raised serum liver enzyme were randomized to receive TDF or placebo for 3 years. Total RNA-sequencing was performed on paired liver biopsies taken before and after the 3-year intervention in 119 patients. They demonstrated that the TDF group achieved a significantly greater reduction in distinct viral integrations (vs. placebo), with 3.28-fold and 1.81-fold decreases in the expressed integrations per million reads, respectively (analysis of covariance, *p* = 0.037). Additionally, viral integrations significantly correlated with host gene dysregulation. The researchers concluded that inhibition of viral replication reduces the number of transcriptionally active distinct HBV-host DNA integrations in patients with substantial viremia [48].

## 7. Other Considerations in the Case of Early Antiviral Treatment

Despite the growing body of sound scientific evidence supporting early treatment consideration, one also needs to consider the challenges associated with this strategy. 

### 7.1. Efficacy and Safety

Although highly effective in achieving viral suppression in patients with active hepatitis, the currently available NUCs perform less well in achieving viral suppression (and functional cure) in patients with chronic infection. Patients in this disease phase have been shown to be difficult to treat. The HBeAg seroconversion rate and HBV DNA suppression (<20 IU/mL) at 48 weeks was only 3% and 23%, respectively, after 8 weeks of ETV followed by ETV+ PEG IFN among children (median age 10.9 years old) with CHB [49]. Similarly disappointing results were observed with long-term treatment in patients receiving TDF, where only 55% had suppressed HBV DNA (<69 IU/mL) and 5% underwent HBeAg seroconversion at 4 years of treatment [50]. Therefore, prolonged treatment duration is anticipated if NUCs are initiated in patients with HBeAg-positive chronic infection.

Additionally, although the current generation of NUCs are well tolerated with minimal adverse effects, compliance in young patients requiring long-term or lifelong therapy may be more arduous. Nonetheless, there are currently several novel antiviral agents in the developmental pipeline with promising data emerging from clinical trials, and it is likely that these new therapies will be used in combination with established antivirals with greater therapeutic effect. Many of these novel therapies either directly target the immune response or are anticipated to impact immunity indirectly through modulation of the viral lifecycle and antigen production. The emergence of these agents and this novel therapeutic approach is expected to contribute to ‘functional cure’ and, consequently, it is anticipated that patients will be offered finite therapies, hence avoiding lifelong treatment regimens [5,6,7]. 

### 7.2. Cost-Effectiveness

The cost of this long-term or of indefinite duration treatment for large numbers of patients, needs to be carefully considered. Using a model that simulates disease progression, Post et al. compared treatment programs for CHB that start at an early stage of the disease to treatment that begins at a later stage. The analysis concluded that early treatment could improve health, reduce premature deaths, and prevent expensive complications, making it highly cost-effective in the long term [51]. A Spanish study estimated the efficiency and clinical impact of antiviral strategies in CHB patients. A Markov model estimated lifetime complications and direct costs in both HBeAg-positive and HBeAg-negative cohorts. Strategy 1 (treatment for 71% of patients diagnosed with CHB) and strategy 2 (full treatment coverage, i.e., 100% of patients), both based on pegylated interferon (peg-IFN) followed by oral TDF or ETV, were compared to no treatment. Strategy 1 increased quality-adjusted life-years (QALY) (3.98 in HBeAg-positive cohort) and strategy 2 up to 5.60 in the HBeAg-positive cohort. The model predicted avoidance of 128 cases of HCC in HBeAg-positive individuals with strategy 1, and up to 181 with strategy 2. The total cost increased up to EUR 102,841 (strategy 1) and EUR 105,408 in HBeAg-positive patients. Overall, a EUR 1581/QALY gained ratio was estimated versus the natural history for both strategies [52].

This was in contrast to a UK systematic review and economic evaluation assessing the cost-effectiveness of non-invasive liver fibrosis tests for treatment decisions in patients with CHB in the UK. Treating all patients without prior fibrosis assessment had an incremental cost-effectiveness ratio (ICER) of GBP 28,137 per additional QALY gained for HBeAg-negative patients, whereas for HBeAg-positive patients, using Fibroscan was the most cost-effective option with an ICER of GBP 23,345. They concluded that for HBeAg-positive patients, using Fibroscan to identify and treat those with ≥F2, was the most cost-effective option [53].

Recently, Kim et al. presented their analysis on the cost-effectiveness of antiviral treatment in adult patients with ‘immune-tolerant’ phase CHB. The researchers designed a Markov model to compare expected costs and QALYs of starting antiviral treatment at the IT-phase (‘treat-IT’) vs delaying the therapy until active hepatitis phase (‘untreat-IT’) in CHB patients over a 20-year horizon. From a healthcare system perspective, the authors showed that the treat-IT strategy with ETV or TDF had an ICER of USD 16,516/QALY, with an annual HCC incidence of 0.73% in the untreat-IT group. With the annual HCC risk ≥ 0.54%, the treat-IT strategy was cost-effective at a willingness-to-pay threshold of USD 20,000/QALY. They concluded that initiating antiviral therapy in IT phase is cost-effective compared with delaying the treatment until the active hepatitis phase in CHB patients, especially with increasing HCC risk, decreasing drug costs, and consideration of productivity loss [54].

### 7.3. Special Populations

NUCs should be considered in certain subgroups to reduce the risk of HBV transmission even if the patient is in the phase of HBeAg-positive chronic infection. Firstly, pregnant mothers with HBV DNA > 200,000 IU/mL should be given NUCs from the second trimester throughout the pregnancy until postpartum to reduce risk of vertical transmission [5,6,7]. Secondly, healthcare workers who take part in exposure-prone procedures should be treated to suppress serum HBV DNA levels below a certain threshold (range from 200 to 20,000 IU/mL in various guidelines and consensuses) to prevent horizontal transmission [55].

## 8. Conclusions

HBeAg-positive chronic infection is not as benign as the old name ‘immune tolerant’ suggested. There is an emerging body of evidence demonstrating that the integration of HBV DNA into the host genome is initiated during the HBeAg-positive phase, and that events associated with tumorigenesis are already underway in this early phase of the disease. Antiviral treatment starting in the HBeAg-positive chronic infection phase can minimize the intensity and duration of active immune-mediated hepatic inflammation by suppressing HBV DNA replication, and thereby attenuating hepatocyte turnover and the selective pressure for clonal expansion of hepatocytes. Additionally, early antiviral treatment may also reduce the risk of host genome integration of HBV DNA. Although long-term treatment is expected for HBeAg-positive chronic infection, concerns over toxicity associated with long-term or lifelong treatment are alleviated with the current first-line NUCs. Recent economic analyses would also suggest that considering broadening treatment criteria to initiate treatment early in the course of the disease ultimately alleviates the cost of lifelong management of CHB, although it is recognized that this strategy may be challenging for healthcare systems with limited resources. These findings, alongside the possibility of a functional cure, were endorsed by the EASL, such that in its latest guidance, they recommend antiviral treatment for patients with HBeAg-positive chronic infection >30 years old, even if they do not meet the classical treatment criteria. The EASL will hopefully lead the way in removing barriers to treatment in CHB and in ultimately adopting a lower treatment threshold, particularly when considering that data supporting this strategy are now emerging at pace. 

## Figures and Tables

**Table 1:** International guidance on the management of HBeAg-positive chronic infection.

	Definition	Treatment Recommendation
EASL (2017)	HBsAg high. ALT normal. HBV DNA > 10^7^ IU/mL. None/minimal liver disease.	Monitor every 3–6 months. May treat if >30 years even if ALT and biopsy normal. Consider treatment if patient has extrahepatic manifestations of HBV or a family history of HCC/cirrhosis.
AASLD (2018)	HBsAg present for ≥ 6 months. Normal or minimally elevated ALT and/or AST. HBV DNA levels are very high (typically > 10^6^ IU/mL). Liver biopsy or noninvasive test results showing no fibrosis and minimal inflammation.	Monitor at least every 6 months. Consider treatment if >40 years and biopsy shows significant necroinflammation/fibrosis even if ALT persistently borderline normal.
APASL (2016)	ALT persistently normal. Elevated HBV DNA. Absence of significant inflammation or fibrosis on biopsy.	Monitor every 3–6 months. Exclude other causes if elevated ALT. Assess fibrosis non-invasively. Individualize liver biopsy. Treat if evidence of significant inflammation or fibrosis on biopsy. May treat if family history of cirrhosis and/or HCC.

Note: HBeAg, hepatitis B ‘e’ antigen; ALT, alanine aminotransferase; AST, aspartate aminotransferase; HBsAg, hepatitis B surface antigen; HBV DNA, hepatitis B viral load; HCC, hepatocellular carcinoma.
